# COVID-19 Vaccine Preferences in General Populations in Canada, Germany, the United Kingdom, and the United States: Discrete Choice Experiment

**DOI:** 10.2196/57242

**Published:** 2024-10-16

**Authors:** David Salisbury, Jeffrey V Lazarus, Nancy Waite, Clara Lehmann, Sumitra Sri Bhashyam, Marie de la Cruz, Beth Hahn, Matthew D Rousculp, Paolo Bonanni

**Affiliations:** 1 Programme for Global Health, Royal Institute of International Affairs Chatham House London United Kingdom; 2 Graduate School of Public Health and Health Policy (CUNY SPH) City University of New York New York, NY United States; 3 Barcelona Institute for Global Health (ISGlobal), Hospital Clinic University of Barcelona Barcelona Spain; 4 School of Pharmacy University of Waterloo Ontario, ON Canada; 5 Department of Internal Medicine Medical Faculty and University Hospital Cologne University of Cologne Cologne Germany; 6 ICON Insights, Evidence and Value—Patient Centered Outcomes Reading United Kingdom; 7 ICON Insights, Evidence and Value—Patient Centered Outcomes Raleigh, NC United States; 8 Novavax, Inc Gaithersburg, MD United States; 9 Department of Health Services University of Florence Florence Italy

**Keywords:** COVID-19, discrete choice experiment, vaccine hesitancy, vaccine side effects, SARS-CoV-2, COVID vaccination, immunization, preference elicitation, informed decision-making, antivaccine

## Abstract

**Background:**

Despite strong evidence supporting COVID-19 vaccine efficacy and safety, a proportion of the population remains hesitant to receive immunization. Discrete choice experiments (DCEs) can help assess preferences and decision-making drivers.

**Objective:**

We aim to (1) elicit preferences for COVID-19 vaccines in Canada, Germany, the United Kingdom, and the United States; (2) understand which vaccine attributes people there value; and (3) gain insight into the choices that different population subgroups make regarding COVID-19 vaccines.

**Methods:**

Participants in the 2019nCoV-408 study were aged ≥18 years; self-reported antivaccinationists were excluded. A DCE with a series of 2 hypothetical vaccine options was embedded into a survey to determine participant treatment preferences (primary objective). Survey questions covered vaccine preference, previous COVID-19 experiences, and demographics, which were summarized using descriptive statistics to understand the study participants’ backgrounds. In the DCE, participants were provided choice pairs: 1 set with and 1 without an “opt-out” option. Each participant viewed 11 unique vaccine profiles. Vaccine attributes consisted of type (messenger RNA or protein), level of protection against any or severe COVID-19, risk of side effects (common and serious), and potential coadministration of COVID-19 and influenza vaccines. Attribute level selections were included for protection and safety (degree of effectiveness and side effect risk, respectively). Participants were stratified by vaccination status (unvaccinated, or partially or fully vaccinated) and disease risk group (high-risk or non–high-risk). A conditional logit model was used to analyze DCE data to estimate preferences of vaccine attributes, with the percentage relative importance calculated to allow for its ranking. Each model was run twice to account for sets with and without the opt-out options.

**Results:**

The mean age of participants (N=2000) was 48 (SD 18.8) years, and 51.25% (1025/2000) were male. The DCE revealed that the most important COVID-19 vaccine attributes were protection against severe COVID-19 or any severity of COVID-19 and common side effects. Protection against severe COVID-19 was the most important attribute for fully vaccinated participants, which significantly differed from the unvaccinated or partially vaccinated subgroup (relative importance 34.8% vs 30.6%; *P*=.049). Avoiding serious vaccine side effects was a significantly higher priority for the unvaccinated or partially versus fully vaccinated subgroup (relative importance 10.7% vs 8.2%; *P*=.044). Attributes with significant differences in the relative importance between the high-risk versus non–high-risk subgroups were protection against severe COVID-19 (38.2% vs 31.5%; *P*<.000), avoiding common vaccine side effects (12% vs 20.5%; *P*<.000), and avoiding serious vaccine side effects (9.7% vs 7.5%; *P*=.002).

**Conclusions:**

This DCE identified COVID-19 vaccine attributes, such as protection against severe COVID-19, that may influence preference and drive choice and can inform vaccine strategies. The high ranking of common and serious vaccine side effects suggests that, when the efficacy of 2 vaccines is comparable, safety is a key decision-making factor.

## Introduction

The World Health Organization (WHO) issued its first emergency use authorization for a COVID-19 vaccine on December 31, 2020 [1], followed by a total of 12 emergency use authorizations for vaccines by the end of 2022 [2]. Despite medical evidence of the importance and safety of these vaccines, a proportion of the public remains hesitant and/or opposed to COVID-19 vaccination. Understanding the public’s preferences for COVID-19 vaccines and drivers of vaccine hesitancy is critical for implementing effective strategies to increase vaccine uptake within the context of a dynamic viral and regulatory landscape [3,4].

To mitigate “COVID-19 fatigue,” vaccine disinformation, and vaccine hesitancy, the WHO Emergency Committee emphasized the need for social media listening and community engagement to assist in tailoring public communications surrounding disease risk and to contextualize evolving health policies [4]. Providing decision makers with quantitative insights into vaccine hesitancy, the reasons why people choose some vaccines over others, and how these may differ across countries and population subgroups will help tailor public health messaging and dissemination of information. Additionally, remaining aware of differences in regional approvals and population makeup will assist in developing the most effective vaccine strategies for specific geographies and people.

A discrete choice experiment (DCE) is an assessment method that can elicit and quantify preferences and drivers of decision-making. Various DCEs related to COVID-19 vaccines were conducted early during the pandemic (August 2020-June 2021); however, these were typically within a specific country and/or were not assessed by predefined subpopulations [5-11]. Furthermore, 1 DCE with participants from Europe (Germany, Italy, Spain, and the United Kingdom) and India reported notable differences in preference of the type of vaccine (messenger RNA, mRNA vs protein) and trust in information from their government among the 2 regions [6]. The authors concluded that this highlighted the importance of developing region-specific vaccine strategies. A United States study found that participants were more likely to opt out of vaccination for children, compared with adults, most specifically younger children (aged 0–5 years) [10].

The aim of the 2019nCoV-408 study, designed as a DCE, was to (1) elicit preferences for COVID-19 vaccines in Canada, Germany, the United Kingdom, and the United States; (2) understand which vaccine attributes people in these countries value; and (3) gain insight into the choices that different population subgroups make regarding COVID-19 vaccines. Subpopulations of participants were assessed based on vaccination status and risk of COVID-19. Furthermore, as some national immunization policies include seasonal COVID-19 vaccine recommendations that align with those for the seasonal influenza vaccine [12,13], participants were also asked about the potential dual administration of these 2 vaccines.

## Methods

### Study Design

The survey and DCE elements were developed based on best practices [14] and conducted in 3 stages before finalization [15] and full deployment in stage 4 (Figure S1 in Multimedia Appendix 1). In short, a literature review, social listening, and consultation with key opinion leaders in the COVID-19 vaccine field were used to identify and confirm concepts to be assessed. Usability and functionality were tested by this study’s team at each stage. Participants completed the web-based draft survey during the qualitative phase (stage 1), in addition to there being a soft launch during phase 2, to ensure that the DCE design was performing as expected and that the survey was appropriate for full fielding. The final, open survey was hosted by a web-based global platform (Forsta) and was available to a convenience sample of participants within their platform. IP addresses were used, and participants were provided a unique URL or sent an automatically generated new link once an individual survey was initiated, to track completion. To help prevent duplicate entries, once a respondent completed the survey (or was disqualified from the survey) their IP address was blocked from future entries for the full project duration. Cookies were not assigned.

**Figure 1:**
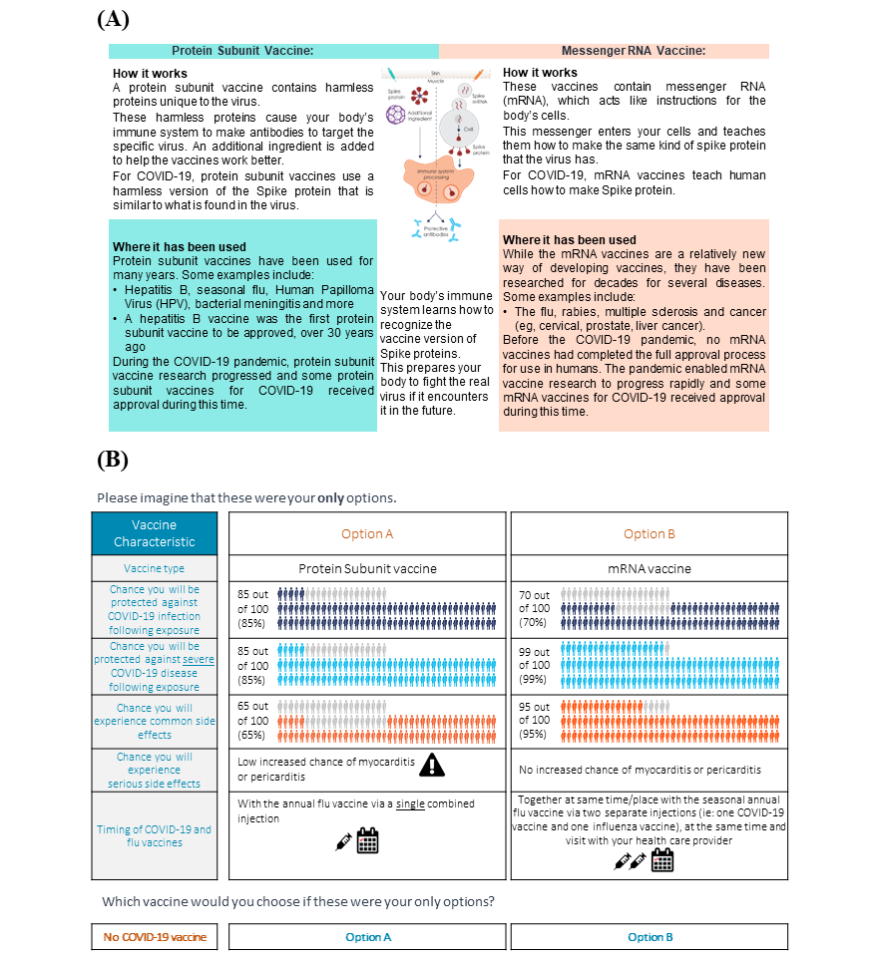
Survey and DCE content. The survey included (A) an infographic overviewing details of the 2 types of COVID-19 vaccines that would appear in the survey and DCE questions and (B) an example presentation of a DCE choice task. DCE: discrete choice experiment; HPV: human papillomavirus; mRNA: messenger RNA.

The questionnaire consisted of 5 sections (screening questions, DCE, vaccine preferences and additional questions, medical history and COVID-19 experience, and sociodemographics) and was anticipated to take 30 min to complete. There were no more than 4 survey questions per page, and there was 1 DCE scenario per page across a total of 65 pages. A brief introduction to this study, an infographic to describe types of COVID-19 vaccines (Figure 1A), and instructions for how to complete the DCE were provided at the start of the survey, as well as practice choice tasks to ensure participant understanding. Logic or quality tests were used to assess the quality of the DCE and the responses provided (and, thus, overall data quality) and included a consistency test, straight-lining choice behavior (always choosing the same option, A or B), distribution of response time, and DCE comprehension questions. Regarding the consistency test, participants who provided different responses to an original and repeat task were considered as having provided inconsistent responses. Appropriate data quality was considered achieved if <15% (n=330) of participants failed any of the logic or quality tests. Respondents with survey completion times in the top and bottom 10% were removed to exclude those with the fastest and slowest response times. The DCE concluded with evaluation questions to assess participant-reported ease of completion. Participants were not able to navigate “back” to adjust submitted responses, and there was no summary display of responses upon completion. Only completed questionnaires were analyzed.

The DCE consisted of choice tasks that were embedded into the survey for fielding. The choice task design was generated using Ngene (version 1.2.1; ChoiceMetrics) software. There were a total of 120 choice tasks that were broken down into 10 blocks of 12, with participants randomized to 1 of the 10 blocks; each participant viewed 11 unique vaccine profiles. Vaccine options varied based on attributes of the vaccine, including type (mRNA vs protein), efficacy (protection from any or severe COVID-19), safety (reduction in common or serious side effects), and potential coadministration of COVID-19 and influenza vaccines (Table S1 in Multimedia Appendix 1). Examples of common side effects included injection-site soreness or swelling, headache, fever, nausea, and muscle aches. Serious side effects considered for this survey were myocarditis and pericarditis. The 2 efficacy attributes—chance of protection from infection and chance of protection from severe disease—were nested to avoid implausible combinations. Attribute-level options were also provided related to the degree of effectiveness and side-effect risk. Participants were asked to respond to each block 2 times: once with 2 hypothetical COVID-19 vaccine options plus an opt-out option (Figure 1B), and once with the opt-out option removed.

### Population

Survey participants were recruited electronically (ie, by email or web-based postings) to complete the open survey using a market research agency and their proprietary participant panel, databases, and consumer recruiter networks, which include patient and physician databases and consumer panels. Template recruitment messages can be seen in Multimedia Appendix 2. Eligible participants were aged ≥18 years; residing in Canada, Germany, the United Kingdom, or the United States; able to read and write English (Canada, the United Kingdom, and the United States) or German (Germany); and had access to the internet via a laptop, desktop, or tablet. People with a cognitive or visual impairment that would impede participation or who self-identified as an “anti-vaccinationist” in specific questions during screening were excluded. Although not formal targets, this study aimed to recruit a diverse sample of participants based on gender (approximately evenly split between male and female), age (approximately even numbers within 10-year brackets), ethnicity (minority representation in line with population demographics), and geography (approximately even representation across countries and geographies within individual countries).

Recruitment was stratified by country, vaccination status (fully vaccinated vs unvaccinated or partially vaccinated), and risk of COVID-19 disease (high-risk vs non–high-risk). Fully vaccinated was defined as participants who believed they were fully vaccinated, having received the initial primary series and additional COVID-19 booster doses. Unvaccinated or partially vaccinated consisted of those who did not receive all primary series or booster doses available to them. Participants were categorized into the protocol-defined high-risk subgroup if they responded that they were considered at an increased risk of COVID-19 due to an existing condition, treatment for a medical condition, or aged ≥60 years. Any participants not considered high-risk were included in the non–high-risk subgroup.

### Statistical Analysis

Enrollment of up to 2000 participants was planned, approximately 500 from each country. While no formal sample size calculation was available for DCE studies without prior coefficient data available, the sample size was informed by the International Society for Pharmacoeconomics and Outcomes Research (ISPOR) Conjoint Analysis Experimental Design Good Research Practices Task Force recommended a minimum sample size of 300 participants [16]. Subgroups from each country consisted of participants at an approximately 1:1 ratio based on vaccination status (vaccinated:unvaccinated or partially vaccinated) and COVID-19 disease risk status (high-risk:non–high-risk). The DCE was developed, fielded, and analyzed according to best practices [17]. Demographic data and survey responses are presented using descriptive summary statistics.

DCE survey data were analyzed using multinomial and mixed logit models to evaluate the impact of each vaccine attribute (independent variable) on participant choices (dependent variable). The relative importance of individual vaccine attributes was compared among subpopulations of the stratification groups (ie, vaccination status and risk group). The relative importance of each attribute was expressed as a percentage weight, calculated by dividing the difference in coefficients for that attribute by the sum of the differences in coefficients for all attributes. Coefficients obtained from the logit model provided an estimate of the log odds ratio of preference for specific vaccine attributes. If 0 did not fall within the 95% CI, then there was a 5% chance that the true value was 0, and differences of ≥5% were considered significant (*P*<.05).

### Ethical Considerations

This study was conducted in compliance with the Declaration of Helsinki, the International Conference on Harmonization Guidelines for Good Clinical Practice, Good Epidemiology Practices, and applicable national regulatory requirements. Details on this study’s purpose, principal investigator, survey duration, and data storage were described in the informed consent form. All persons provided informed consent before participation and were free to withdraw at any time. All participant names and identifiable information were omitted from reports, and each participant had a unique participant identification number. Data protection and privacy of data were regulated and managed according to the European Union General Data Protection Regulation (GDPR). Any materials containing information to identify participants were stored on a GDPR and Health Insurance Portability and Accountability Act (HIPPA)–compliant secure server. Electronic data were deleted from portable devices after the interview analysis was completed. This survey was exempt from review in Germany and the United Kingdom and was approved by a central institutional review board (Salus IRB; protocol 2088-0055) in the United States and Canada on April 6, 2023. There were no known risks to participants. Honoraria were provided to reimburse participants for their time and effort.

## Results

### Population Sociodemographic and Clinical Characteristics

Of the 11,757 unique site visitors, there were 5983 unique visits to the survey page (view rate 50.89%), and 94.02% (5625/5983) agreed to participate. There was a 45.88% (2581/5625) completion rate, with results taken from the first 2000 participants. They (N=2000) were surveyed from July to August 2023. The mean age of the overall population was 47.6 (SD 18.8) years, and 51.25% (1025/2000) identified as male; general demographics were comparable among participants in the unvaccinated or partially and fully vaccinated subgroups (Table S2 in Multimedia Appendix 1). Most (72.1%, 1442/2000) participants resided with another person (eg, spouse, partner, child, or another family member), and approximately half (47.1%, 942/2000) of the population were married or in a civil union. While data on race or ethnicity could not be collected in Germany, the majority (82.4%, 1236/1500) of participants in Canada, the United Kingdom, and the United States were White (Figure S2 in Multimedia Appendix 1).

An undergraduate or postgraduate degree had been obtained by 62% (310/500), 56.6% (283/500), 47.6% (238/500), and 52.4% (262/500) of participants in Canada, Germany, the United Kingdom, and the United States, respectively. The majority (74.6%, 1492/2000) of participants responded that they had no disability or impairment. Most participants submitted that they were on a government or national health insurance plan in the United Kingdom (the National Health Service; 90.2%, 451/500), Germany (statutory health insurance; 86.4%, 432/500), and North America (Canada and the United States, combined; Medicare or Medicaid; 59%, 590/1000); 46.1% (461/1000) of participants in Canada and the United States had private health insurance. Based on the availability of universal coverage in Canada, Germany, and the United Kingdom, all participants in these regions should have submitted responses indicating being on, or eligible for, a national or government health insurance plan; however, some respondents may have only selected 1 option (ie, private insurance) instead of all applicable insurance options. In total, 49.2% (985/2000) of participants had previously had COVID-19. Of these participants, 47.1% (464/985) and 42.2% (416/985) had mild and moderate symptoms, respectively.

### Survey of Perception of COVID-19

**Figure 2:**
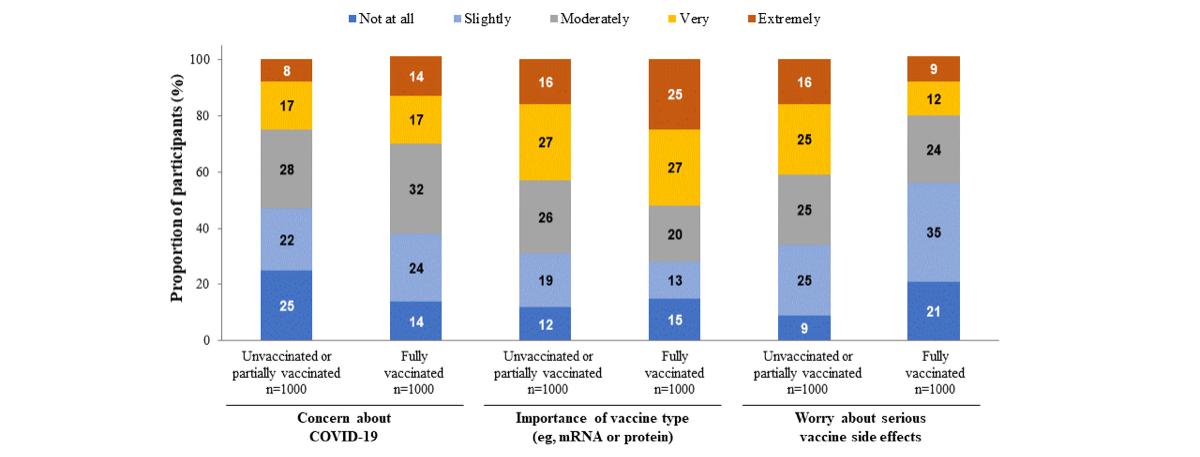
Surveyed perceptions of COVID-19 and vaccine preferences. Responses from all participants (N=2000). mRNA: messenger RNA.

Participants were surveyed on their perception of COVID-19, as well as preferences for various COVID-19 vaccine traits (Table 1 and Figure 2). Some questions included the concept of having more than one vaccine option to choose from; however, it is noteworthy that a choice may not have been available in real-world situations for all participants. Overall, 69.45% (1389/2000) of participants were slightly to very worried about COVID-19, and of participants 55.85% (1117/2000) felt they always adhered to COVID-19 guidelines.

**Table 1:** Survey outcomes for the perception of COVID-19 vaccine preferences.

Participant preference			Unvaccinated or partially vaccinated (n=1000), n (%)		Fully vaccinated (n=1000), n (%)		Total population (N=2000), n (%)
**Concern about COVID-19**							
	Not at all	252 (25)		140 (14)		392 (20)	
	Slightly worried	217 (22)		235 (24)		452 (23)	
	Moderately worried	279 (28)		320 (32)		599 (30)	
	Very worried	170 (17)		168 (17)		338 (17)	
	Extremely worried	82 (8)		137 (14)		219 (11)	
**How often adhere to COVID-19 guidelines**							
	Never	13 (1)		5 (1)		18 (1)	
	Rarely	34 (3)		10 (1)		44 (2)	
	Sometimes	111 (11)		65 (7)		176 (9)	
	Often	345 (35)		300 (30)		645 (32)	
	Always	497 (50)		620 (62)		1117 (56)	
**Importance of getting a choice of COVID-19 vaccines**							
	Not at all important	41 (4)		43 (4)		84 (4)	
	Slightly important	64 (6)		77 (8)		141 (7)	
	Moderately important	231 (23)		223 (22)		454 (23)	
	Very important	370 (37)		317 (32)		687 (34)	
	Extremely important	294 (29)		340 (34)		634 (32)	
**Importance of vaccine type (eg, protein-based or mRNA^a^)**							
	Not important at all	120 (12)		145 (15)		265 (13)	
	A little important	193 (19)		128 (13)		321 (16)	
	Moderately important	261 (26)		204 (20)		465 (23)	
	Quite important	270 (27)		274 (27)		544 (27)	
	Extremely important	156 (16)		249 (25)		405 (20)	
**Importance of the duration of protection**							
	Not important	66 (7)		15 (2)		81 (4)	
	A little important	121 (12)		66 (7)		187 (9)	
	Moderately important	239 (24)		168 (17)		407 (20)	
	Quite important	321 (32)		430 (43)		751 (38)	
	Extremely important	253 (25)		321 (32)		574 (29)	
**Duration of protection preference**							
	Every 12 months	446 (45)		538 (54)		984 (49)	
	Every 6 months	89 (9)		219 (22)		308 (15)	
	No preference	234 (23)		220 (22)		454 (23)	
	Would not want a vaccine	231 (23)		23 (2)		254 (13)	
**Worry about serious vaccine side effects**							
	Not at all worried	90 (9)		212 (21)		302 (15)	
	A little worried	251 (25)		346 (35)		597 (30)	
	Moderately worried	253 (25)		236 (24)		489 (24)	
	Quite worried	245 (25)		120 (12)		365 (18)	
	Extremely worried	161 (16)		86 (9)		247 (12)	
**Would consider getting a single vaccine against both COVID-19 and influenza**							
	Yes	447 (45)		770 (77)		1217 (61)	
	No	142 (14)		69 (7)		211 (11)	
	Would not want a COVID-19 vaccine	63 (6)		13 (1)		76 (4)	
	Would not want an influenza vaccine	65 (7)		30 (3)		95 (5)	
	Would not want either vaccine	72 (7)		8 (1)		80 (4)	
	Do not know or unsure	211 (21)		110 (11)		321 (16)	
**Preferred timing of a COVID-19 vaccine in relation to an influenza vaccine**							
	Different injections at same time or place	232 (23)		277 (28)		509 (25)	
	Single combined injection	200 (20)		350 (35)		550 (28)	
	Separate injections at different time	337 (34)		202 (20)		539 (27)	
	Indifferent	231 (23)		171 (17)		402 (20)	

^a^mRNA: messenger RNA.

Regarding the attributes that influence the decision to receive a COVID-19 vaccine, 66.05% (1321/2000) of people in the overall population, as well as in the fully (65.7%, 657/1000) and unvaccinated or partially (66.4%, 664/1000) vaccinated subgroups, considered the availability of choice of the COVID-19 vaccine to be very or extremely important (Table 1). The type of COVID-19 vaccine (ie, protein subunit or mRNA) was considered moderately-to-extremely important in decision-making by 70.7% (1414/2000) of all participants, with similar proportions occurring in the fully vaccinated (72.7%, 727/1000) and unvaccinated or partially vaccinated (68.7%, 687/1000) subgroups (Table 1, Figure 2). Over half (55.05%, 1101/2000) of participants responded that they were moderately, quite, or extremely worried about serious vaccine side effects, with people in the unvaccinated or partially vaccinated subgroup (65.9%, 659/1000) being more concerned than fully vaccinated people (44.2%, 442/1000).

Establishing a duration of protection was quite-to-extremely important for 66.25% (1325/2000) of all participants, more so for fully than unvaccinated or partially vaccinated participants (75.1%, 751/1000 vs 57.4%, 574/1000, respectively; Table 1). More specifically, establishing a protection period for vaccination to occur every 6 or 12 months was preferred by a greater percentage of people in the fully (75.7%, 757/1000) versus unvaccinated or partially (53.5%, 535/1000) vaccinated subgroups, with 23.1% (231/1000) of all unvaccinated or partially vaccinated participants disclosing that they would not want the vaccine. When asked about considering receipt of a single combined vaccine against both COVID-19 and influenza, 60.85% (1217/2000) of all participants were willing, with a greater proportion of people in the fully (77%, 770/1000) versus unvaccinated or partially (44.7%, 447/1000) vaccinated subgroups considering the combined, single immunization option.

### General Vaccine Preferences

In terms of factors that influence the decision to receive any vaccine (ie, not COVID-19-specific), the majority of respondents considered the length of time that the vaccine was tested in humans; perceived reliability and trustworthiness of vaccine information; length of time that the vaccine was evaluated in a vaccination program; and vaccine type to be important (Figure S3 in Multimedia Appendix 1). Other than the reliability and trustworthiness of vaccine information, results were generally consistent among all participants and the fully and unvaccinated or partially vaccinated subgroups. For vaccine information, 81.7% (817/1000) of those in the fully vaccinated subgroup and 48.3% (483/1000) of unvaccinated or partially vaccinated participants agreed this was important.

### Preferences for COVID-19 Vaccine Attributes

Choice task assessment revealed that the most important COVID-19 vaccine attributes among all participants were protection against severe COVID-19 (relative importance 28%), protection against any severity COVID-19 (26.4%), and common side effects (18.4%). The least important of the attributes presented were potential coadministration (11.1%; eg, together in a single injection, separately at the same time, or injected on different days) of the COVID-19 and influenza vaccines and whether the COVID-19 vaccine was mRNA-based or used a protein subunit (4.6%).

Participant characteristics affected their vaccine preferences. Based on the participant risk group, protection against severe COVID-19 was the most important of the presented attributes for the high-risk subgroup (n=1000), significantly more so compared with the non–high-risk subgroup (n=1000; relative importance 38% vs 32%; *P*<.000). The importance of protection from COVID-19 (any severity) was the next most important and comparable among these 2 risk groups. Avoiding common vaccine side effects was significantly more important to the non–high-risk subgroup than the high-risk subgroup (relative importance 21% vs 12%, *P*<.000). Avoiding serious vaccine side effects was the fourth most important attribute for both subgroups; however, it was significantly more so for those considered high-risk versus non–high-risk (relative importance 10% vs 7%; *P*<.026).

The subgroup of participants who were fully vaccinated responded that protection against severe COVID-19 was the most important attribute, which was significantly different compared with the unvaccinated or partially vaccinated subgroup (relative importance 35% vs 31%; *P*<.049). Protection from any-severity COVID-19 and avoidance of common vaccine side effects were the next most important attributes for both subgroups, with comparable responses. Avoiding serious vaccine side effects was of significantly higher priority for the unvaccinated or partially versus fully vaccinated subgroups (relative importance 11% vs 8%; *P*<.044).

No significant differences in the prioritization of COVID-19 vaccine attributes were seen when participants were stratified by education level (no higher education vs university degree and above) or race (White vs non-White). Evaluation questions indicated that the choice tasks were easy to understand and answer, and selections were relevant to and reflective of real-life decisions (Table S3 in Multimedia Appendix 1).

## Discussion

### Principal Results

This study used a survey and DCE to examine preferences for COVID-19 vaccines in 2 North American (Canada and the United States) and 2 European (Germany and the United Kingdom) countries. These data add to the limited number of DCEs evaluating COVID-19 vaccine preferences in an international cohort [6,18,19]. Among the 6 vaccine attributes assessed in this DCE, results indicated that the highest priorities (overall and based on risk group or vaccination status) were protection against COVID-19 of any severity and protection against severe COVID-19. The chance of common (ie, injection-site soreness or swelling, headache, fever, nausea, and muscle aches) and serious (ie, myocarditis or pericarditis) vaccine side effects were the next most important attributes in all subgroups examined, indicating that when the efficacy of 2 vaccines is comparable, safety is the key decision-making factor.

The importance of common and serious vaccine side effects differed significantly based on population characteristics, such as risk status. Differences in consideration of side effects in the general and high-risk populations, as well as preliminary evidence that protein-based vaccines may have lower reactogenicity (ie, injection-site reactions, fatigue, headache, fever, and nausea) compared with their mRNA counterparts [20], are important during the development of vaccine strategies and communications. A survey on COVID-19 vaccines conducted in 2021 found that transparency on the likelihood of experiencing side effects, combined with reinforcing the benefits of vaccination, can positively impact vaccine interest [21]. It is also important to highlight the mild nature and short duration of side effects that commonly occur after vaccination. Combining this information with data gained on vaccine perception from this DCE can help develop more effective vaccine-related communications to alleviate concerns over vaccine side effects.

Outcomes from the survey questions on COVID-19 vaccine perceptions indicated that there was an overall willingness of participants to consider receipt of influenza and COVID-19 vaccines at the same time. Notably, when presented alongside other options in the DCE, the timing of COVID-19 and influenza vaccination (together or at separate times) was ranked with low importance, compared with the other vaccine attributes presented. Conversely, while most participants responded in the perception survey that vaccine type (mRNA vs protein-based) was moderately-to-extremely important, attribute importance from the DCE for the type of vaccine had values of <3% among subgroups and was the least important of the attributes presented.

### Comparison With Prior Work

In a global study on COVID-19 vaccine acceptance conducted from June to July 2022 (a 30-question survey instrument administered to 23,000 respondents across 23 countries), willingness to receive a COVID-19 vaccine increased by approximately 4% from 2021 to 2022, although 12% of vaccinated survey participants indicated hesitancy toward booster dosing [3]. Reasoning behind the lack of booster uptake may be related to 25.2% of respondents indicating a perceived lower COVID-19 disease severity at the time of the survey. Additionally, the global study revealed that (from 2021 to 2022) almost 2 out of 5 (approximately 40%) participants paid less attention to releases of new COVID-19 information and data and that there was an overall decrease in their support for vaccine mandates. In late 2023, perspectives on COVID-19 and vaccine acceptance were evaluated in another report from this study [22]. The updated survey of 23,000 people reveals their resistance to vaccination, including a decrease in intent to receive a COVID-19 booster among vaccinated participants (from 87.9% in 2022 to 71.6% in 2023). In addition, results suggest that concerns about COVID-19 vaccination have had an impact on perceptions related to vaccination for other diseases; notably, 23.1% of participants reported that they were less willing to be vaccinated for diseases other than COVID-19 as a result of their experience during the pandemic. While regional differences were described for some of the survey questions, no notable differences in these major conclusions were observed among the countries studied. The investigators concluded that vaccine hesitancy and trust challenges remain, emphasizing the need for targeted, culturally sensitive health communication strategies.

Choice experiments, such as the DCE reported here, and conjoint analyses, provide a precise estimation of which attributes people prioritize when considering COVID-19 vaccination and, thus, can help guide what information might be meaningful to communicate. Earlier DCEs that focused on COVID-19 vaccines have been performed in many regions, including those of this study (Canada, Germany, the United Kingdom, and the United States), although most of these studies focused on an individual region or country [5,7-11,23]. Indeed, some of these studies were tailored to understand specific drivers within a region; for example, the importance of where a COVID-19 vaccine was developed or produced [5,8,23]. Updated choice experiments are needed to understand decision-making across multiple regions because perceptions related to COVID-19 vaccination have changed over the years [22] from the early months of vaccine campaigns [24]. Prioritization of protection from disease has been observed consistently in DCEs conducted before 2023 in multiple regions [5-11,23]. Similarly, choice experiments in the same time period using conjoint analysis methodology have shown the importance of vaccine efficacy [25-27]. Our DCE extends these findings by examining preferences for COVID-19 vaccines in 2023 within a large, international study population. In this multicountry sample, similar to previous studies in individual countries [5,7-11,23,25-27] and an earlier multicountry study [6], the highest priority was protection from COVID-19.

Our finding that vaccine side effects were the next most important attribute when choosing a COVID-19 vaccine is also consistent with earlier studies [5,8-11,23,25-27]. In our survey, even common vaccine side effects were important, particularly among non–high-risk participants (vs high-risk) and those who were unvaccinated or partially vaccinated (vs fully vaccinated). Similarly, a survey of over 9000 adults in Japan found that experiencing any local hypersensitivity reactions to COVID-19 vaccines decreased confidence in the safety of the vaccination as well as the probability of taking a second dose of a COVID-19 vaccine [28]. This observation was consistent regardless of prior confidence in vaccine safety or prior vaccine hesitancy. Concerns about safety and the potential adverse impact on willingness to receive a COVID-19 vaccination have been observed in studies using a variety of methodologies in addition to choice experiments [5,8-11,23-27,29-33]. Furthermore, DCEs conducted on immunizations targeting viruses that do not cause COVID-19 also identified side effects as a key factor impacting willingness to receive a vaccine. Additionally, 1 survey reported that whether a participant experienced side effects from the previous years’ influenza vaccine was one of the strongest influences on subsequent influenza vaccine uptake [34]. It is important to note that these comparisons are limited because the studies used different surveys and different methodologies for the choice experiments; however, the general findings are largely consistent.

### Limitations

The large number of respondents (N=2000) in this assessment ensured good precision regarding estimates of attribute importance and allowed vaccine preferences in various population subgroups to be explored. However, survey participants were obtained via convenience sampling and may not fully represent the general population of each country. These analyses relied on self-reported preferences, which may overstate value and willingness [35] and may not always align with decisions and preferences in real-world settings [36,37]. This survey was developed in late 2022, and the DCE incorporated real-world and clinical data available at that time. Given changes in the vaccine landscape and health policy over time, there is potential for temporal bias.

### Conclusions

Overall, this study reports COVID-19 vaccine attributes that may influence preference and drive choice. The information gained from the general population across the 4 study countries may be combined with additional regional data to help develop global and regional vaccine program strategies. Additionally, an improved understanding of the importance of vaccine side-effect perceptions (specifically related to the COVID-19 risk group and to feelings of vaccine hesitancy) can help develop more relevant messaging to inform the population on various vaccine characteristics.

## Appendix

Multimedia Appendix 1 Material showing hypothetical COVID-19 vaccine attributes and associated levels (Table S1), sociodemographic characteristics by vaccination status or country (Table S2), outcomes of DCE evaluation questions (Table S3), survey and DCE development schema (Figure S1), participant race or ethnicity and health insurance status by vaccination status and country (Figure S2), and importance of attributes on the decision to receive any vaccine in different subgroups (Figure S3). DCE: discrete choice experiment.

Multimedia Appendix 2 File describing template recruitment messages.

## Abbreviations

DCE: discrete choice experiment
mRNA: messenger RNA
WHO: World Health Organization

## Data Availability

The data generated and analyzed during this study are available from the corresponding author upon reasonable request.
