# Supplementary material for: COVID-19 Vaccine Preferences in General Populations in Canada, Germany, the United Kingdom, and the United States: Discrete Choice Experiment
Source: JMIR Public Health Surveill. 2024 Oct 16;10:e57242. doi: 10.2196/57242 (PMC11525078; doi:10.2196/57242)
Supplement: Multimedia Appendix 1 [file publichealth_v10i1e57242_app1.docx]

**MULTIMEDIA APPENDIX 1**

**Table S1.** Hypothetical COVID-19 vaccine attributes and associated levels

| **Attribute name** | **Patient-facing attribute levels** |
| --- | --- |
| Vaccine type | - Protein subunit vaccine - mRNA vaccine |
| Chance you will be protected against COVID-19 infection following exposure | - 99% - 85% - 70% - 55% |
| Chance you will be protected against severe COVID-19 disease following exposure | - 99% - 85% - 70% - 55% |
| Chance you will experience common side effects | - 95% - 80% - 65% - 50% |
| Chance you will experience serious side effects | - Low increased chance of myocarditis or pericarditis - No increased chance of myocarditis or pericarditis |
| Timing of COVID-19 and flu vaccines | - Single combined injection - Separate injections, but at the same time/place - Separate injections at a different time |

**Table S2.** Sociodemographic characteristics by vaccination status and/or country.

|  | | **Unvaccinated or partially vaccinated** | **Fully  vaccinated** | **Total  population** |
| --- | --- | --- | --- | --- |
| **Age, mean (SD)** | | 45.4 (17.2) | 49.9 (19.9) | 47.6 (18.8) |
| **Gender, n (%)** | | n=1000 | n=1000 | N=2000 |
|  | Male | 476 (48) | 549 (55) | 1025 (51) |
|  | Female | 523 (52) | 444 (44) | 967 (48) |
|  | Transgender | 0 | 2 (<1) | 2 (<1) |
|  | Do not identify | 1 (<1) | 5 (1) | 6 (<1) |
| **Marital status, n (%)** | | n=1000 | n=1000 | N=2000 |
|  | Married or in a civil union/partnership | 444 (44) | 498 (50) | 942 (47) |
|  | Single/widowed | 286 (29) | 287 (29) | 573 (29) |
|  | In a relationship (living together or apart) | 120 (12) | 71 (7) | 191 (10) |
|  | Divorced/separated | 92 (9) | 65 (7) | 157 (8) |
|  | Missing/prefer not to answer | 58 (6) | 79 (8) | 137 (7) |
| **Living situation, n (%)** | | n=1000 | n=1000 | n=2000 |
|  | With a partner/spouse | 420 (42) | 488 (49) | 908 (45) |
|  | Alone | 232 (23) | 251 (25) | 483 (24) |
|  | With children | 188 (19) | 135 (14) | 323 (16) |
|  | With other family members | 120 (12) | 91 (9) | 211 (11) |
|  | House-share or flat-share | 33 (3) | 28 (3) | 61 (3) |
|  | Prefer not to answer | 7 (1) | 6 (1) | 13 (1) |
|  | Care/nursing home | 0 | 1 (<1) | 1 (<1) |
| **Employment status, n (%)** | | n=1000 | n=1000 | N=2000 |
|  | Working full-time | 449 (45) | 414 (41) | 863 (43) |
|  | Retired | 220 (22) | 338 (34) | 558 (28) |
|  | Working part-time | 108 (11) | 83 (8) | 191 (10) |
|  | Self-employed | 60 (6) | 48 (5) | 108 (5) |
|  | Student | 49 (5) | 45 (5) | 94 (5) |
|  | Unemployed | 54 (5) | 34 (3) | 88 (4) |
|  | Unable to work due to sickness or injury | 29 (3) | 21 (2) | 50 (2) |
|  | Looking after home or family | 28 (3) | 14 (1) | 42 (2) |
|  | Prefer not to answer | 3 (<1) | 3 (<1) | 6 (<1) |
| **Disability/Impairment, n (%)** | | n=1000 | n=1000 | N=2000 |
|  | None | 733 (73) | 759 (76) | 1492 (75) |
|  | Mobility impairment | 78 (8) | 73 (7) | 151 (8) |
|  | Mental health disorder | 90 (9) | 58 (6) | 148 (7) |
|  | Vision/hearing impairment | 67 (7) | 78 (8) | 145 (7) |
|  | Other (not listed) | 49 (5) | 41 (4) | 90 (5) |
|  | Learning disability | 28 (3) | 28 (3) | 56 (3) |
|  | Prefer not to answer | 26 (3) | 13 (1) | 39 (2) |
| **Education, n (%)** | |  |  |  |
| **Canada** | | n=250 | n=250 | n=500 |
|  | College, CÉGEP, or other non-university diploma | 61 (24) | 61 (24) | 122 (24) |
|  | Bachelor’s degree | 52 (21) | 70 (28) | 122 (24) |
|  | High (secondary) school or equivalent | 67 (27) | 54 (22) | 121 (24) |
|  | Post-graduate degree | 26 (10) | 40 (16) | 66 (13) |
|  | Apprenticeship or trades diploma | 22 (9) | 16 (6) | 38 (8) |
|  | Less than high (secondary) school | 22 (9) | 8 (3) | 30 (6) |
|  | Prefer not to answer | 0 | 1 (<1) | 1 (<1) |
| **Germany** | | n=250 | n=250 | n=500 |
|  | Work-study program (vocational or commercial school) completed or equivalent | 85 (34) | 56 (22) | 141 (28) |
|  | College degree (bachelor’s, master’s, etc.) | 49 (20) | 52 (21) | 101 (20) |
|  | European baccalaureate or equivalent | 49 (20) | 41 (16) | 90 (18) |
|  | Polytechnic college degree | 16 (6) | 33 (13) | 49 (10) |
|  | Tenth grade diploma or equivalent | 25 (10) | 21 (8) | 46 (9) |
|  | Doctorate or habilitation | 12 (5) | 31 (12) | 43 (9) |
|  | Secondary school diploma | 14 (6) | 14 (6) | 28 (6) |
|  | No formal qualification | 0 | 2 (1) | 2 (<1) |
| **United Kingdom** | | n=250 | n=250 | n=500 |
|  | Undergraduate degree | 86 (34) | 84 (34) | 170 (34) |
|  | GCSEs/nationals or equivalent | 40 (16) | 45 (18) | 85 (17) |
|  | Vocational/technical qualification | 42 (17) | 43 (17) | 85 (17) |
|  | Advanced subsidiary/ advanced level/Scottish Highers or equivalent | 50 (20) | 30 (12) | 80 (16) |
|  | Post-graduate degree | 27 (11) | 41 (16) | 68 (14) |
|  | No formal qualification | 5 (2) | 7 (3) | 12 (2) |
| **United States** | | n=250 | n=250 | n=500 |
|  | Bachelor’s degree | 74 (30) | 90 (36) | 164 (33) |
|  | Post-graduate degree | 35 (14) | 63 (25) | 98 (20) |
|  | Completed high school or GED | 49 (20) | 35 (14) | 84 (17) |
|  | Some college | 51 (20) | 28 (11) | 79 (16) |
|  | Associate degree | 25 (10) | 25 (10) | 50 (10) |
|  | Vocational/technical training | 14 (6) | 5 (2) | 19 (4) |
|  | Some high school | 2 (1) | 4 (2) | 6 (1) |

CÉGEP, collège d'enseignement general et professionnel (general and professional teaching college); GCSE, general certificate of secondary education; GED, general educational development.

**Table S3.** Outcomes of DCE evaluation questions.

| **DCE Evaluation Question** | **Mean (SD)** |
| --- | --- |
| Participants understanding of the scenarios on a scale from 1 (“Did not understand the scenarios at all”) to 10 (“Completely understood the scenarios”) | 8.80 (1.21) |
| Participants ease of understanding the scenarios on a scale from 1 (“Very Difficult”) to 10 (“Very Easy”) | 8.22 (1.60) |
| How well participants think their choices for each scenario reflect what they would do in real life (1= “Not at all” and 10= “Completely) | 8.32 (1.60) |
| How relevant participants found the scenarios to their current situation (1= “Not at all” and 10= “Completely) | 7.74 (2.08) |

DCE, discrete choice experiment; SD, standard deviation.

**
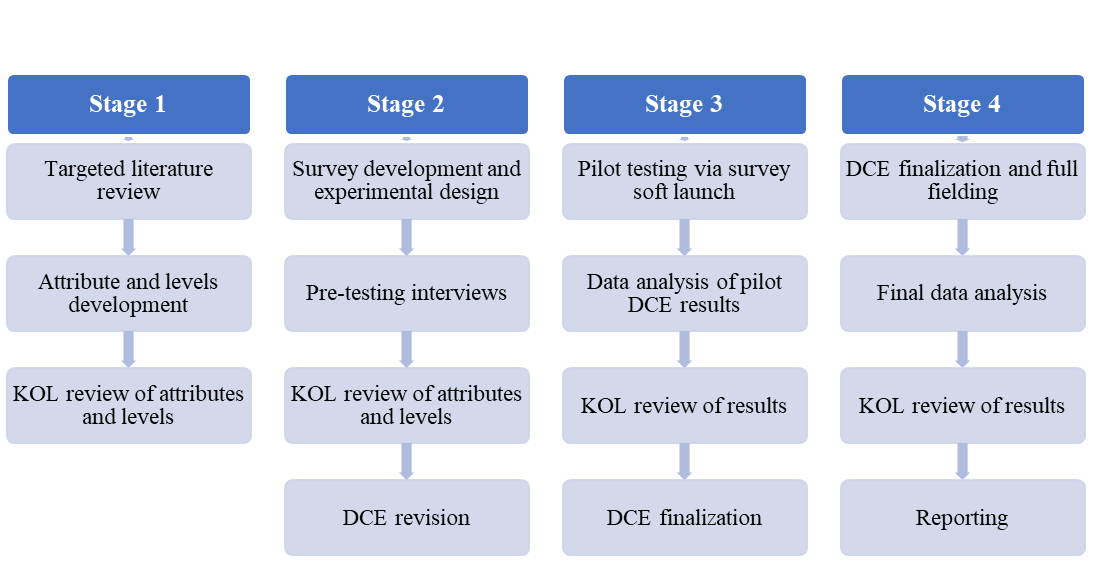
**

**Figure S1.** Survey and DCE development schema.

DCE, discrete choice experiment; KOL, key opinion leader.

**Figure S2.** Participant race/ethnicity and health insurance status by vaccination status and country.

^a^Data on race/ethnicity cannot be collected in Germany; percentages may total >100 due to participants being able to select multiple race/ethnicity options. Other includes Native American or Alaskan Native and Native Hawaiian or other Pacific Islander. ^b^Responses for government plans in Canada, Germany, and the UK should be 100% based on the availability of universal coverage; however, some respondents may have only selected one option (ie, private insurance) and not all applicable insurance options. UK, United Kingdom; US, United States; vax, vaccinated.

**(A)**


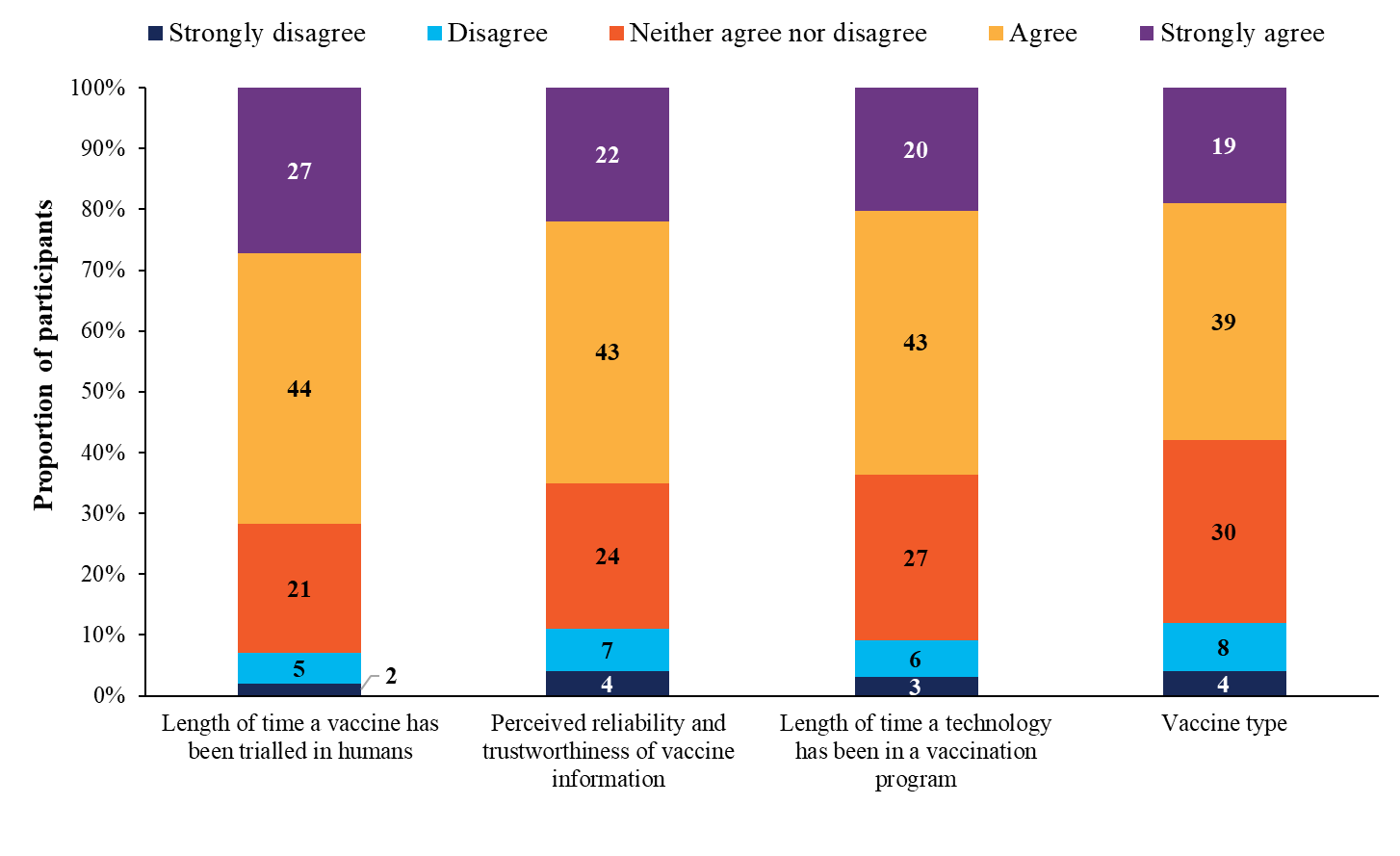


**(B)**


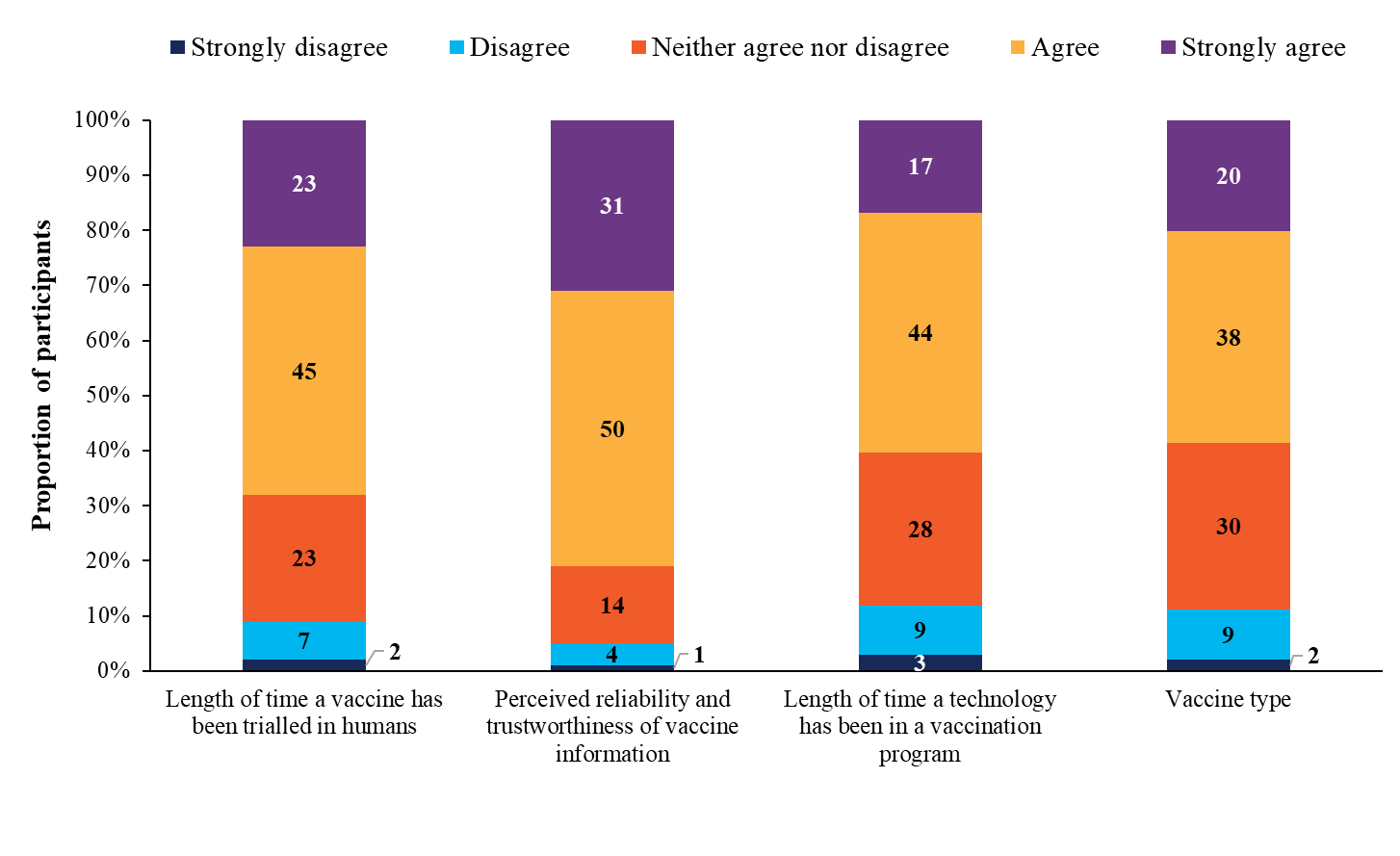


**(C)**


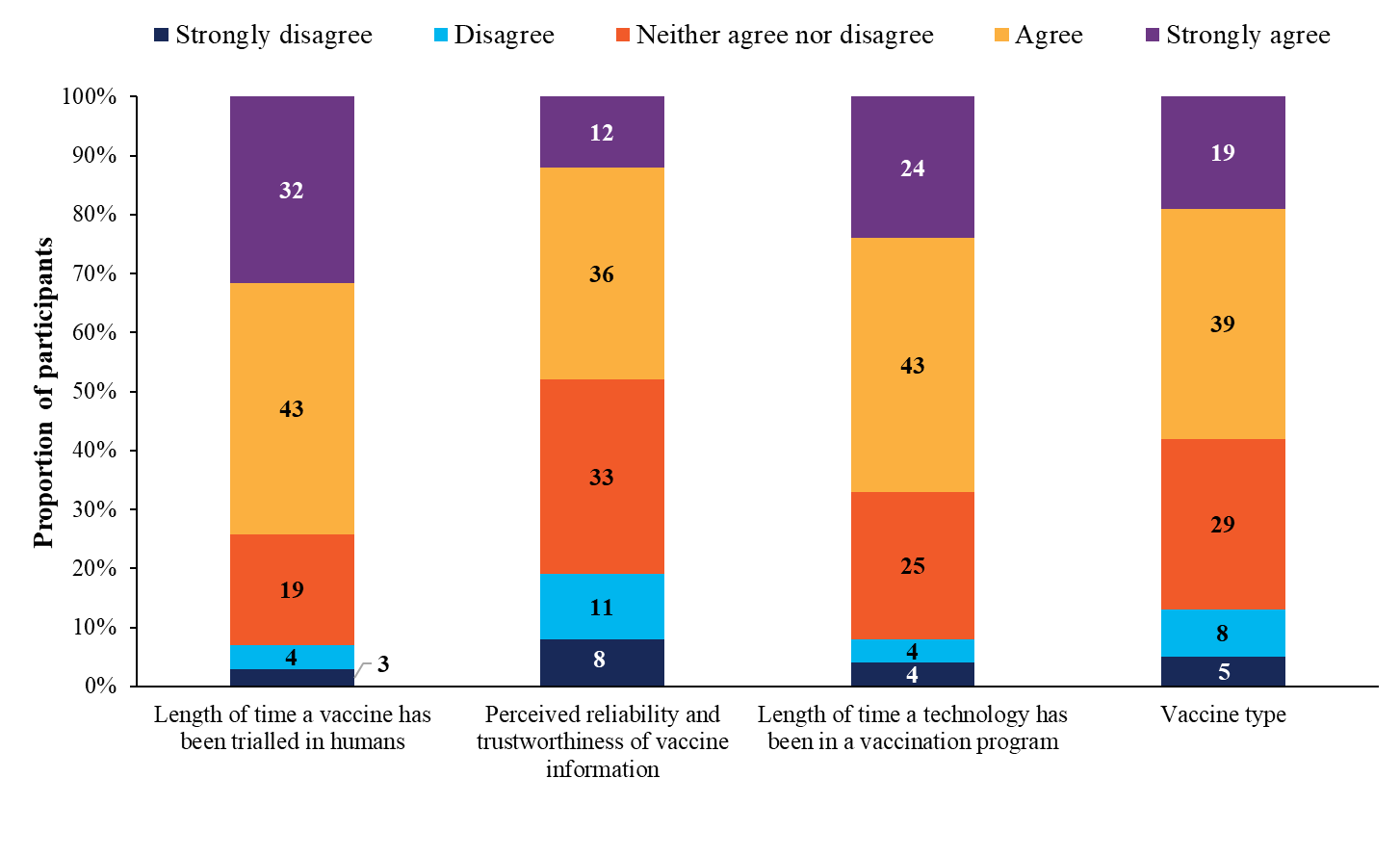


**Figure S3.** Importance of attributes on the decision to receive any vaccine in (A) all participants (N=2000) and participants in the (B) fully (n=1000) and (C) un/partially (n=1000) vaccinated subgroups.
