# Supplementary material for: COVID-19 Vaccine Preferences in General Populations in Canada, Germany, the United Kingdom, and the United States: Discrete Choice Experiment
Source: JMIR Public Health Surveill. 2024 Oct 16;10:e57242. doi: 10.2196/57242 (PMC11525078; doi:10.2196/57242)
Supplement: Multimedia Appendix 2 [file publichealth_v10i1e57242_app2.docx]

Recruitment Messages

**PHASE 1: PRE-TESTING INTERVIEWS**

**1.0) Recruitment ANNOUNCEMENT for an Interview Study – OPTION 1:**

[Global Perspectives / Global Patients / Global Expert] is currently looking for adults to participate in an upcoming paid interview study in [the US / the UK / Germany / Canada / Italy].

The purpose of this study is to better understand attitudes, perceptions, and preferences in relation to COVID-19 vaccines. Eligible participants will be invited to attend a telephone or web-assisted interview that will last approximately 60 minutes in total. Participants will be compensated [100 USD / 65 GBP / 90 EUR / 80 CAD / 80 EUR] for their time.

If you are interested in participating or would like more information about the study, please contact [Contact details].

- 1. **Recruitment ANNOUNCEMENT for an Interview Study – OPTION 2:**

[Global Perspectives / Global Patients / Global Expert] is currently looking for adults to participate in an upcoming paid interview study in [the US / the UK / Germany / Canada / Italy] regarding COVID-19.

Eligible participants will be invited to attend a telephone or web-assisted interview that will last approximately 60 minutes in total. Participants will be compensated [100 USD / 65 GBP / 90 EUR / 80 CAD / 80 EUR] for their time.

If you are interested in participating or would like more information about the study, please contact [Contact details].

**1.2) TO ADMINS ON SOCIAL MEDIA LONG VERSION:**

Good [morning/afternoon/evening],

My name is [recruiter’s name] and I work for [Global Perspectives / Global Patients / Global Expert], where we look for people to take part in scientific studies.

We are looking for adults in [the US / the UK / Germany / Canada / Italy] to participate in an upcoming paid interview study.

The reason for my message is to know if you, as the [Group Administrator/Group Moderator] on [Social Media Channel], would allow me to post a recruitment message on the group wall in case there is someone interested in participating.

The study will consist of a 60min telephone or web-assisted interview. These interviews are designed to explore attitudes, perceptions, and preferences in relation to COVID-19 vaccines.

We appreciate the time that participants invest in our studies and, for this reason, will offer an incentive of [100 USD / 65 GBP / 90 EUR / 80 CAD / 80 EUR] for taking part in the interview as a token of appreciation.

If you need more information, please do not hesitate to contact me via Facebook or email at [Contact details].

Thank You!

**1.3)** **TO ADMINS ON SOCIAL MEDIA SHORT VERSION – OPTION 1:**

Hi [name],

At [Global Perspectives / Global Patients / Global Expert] we are looking for adults to participate in an upcoming paid interview study in [the US / the UK / Germany / Canada/ Italy].

Eligible participants will be invited to attend a telephone or web-assisted interview that will last approximately 60 minutes in total. Participants will be compensated with [100 USD / 65 GBP / 90 EUR / 80 CAD / 80 EUR] for their time.

As a moderator of a [social media/group/page], I am hoping that you would help me take this information to those who might be interested by sharing it or letting me post it in the group you moderate.

If you have any questions, please contact me at [Contact details].

**1.4)** **TO ADMINS ON SOCIAL MEDIA SHORT VERSION – OPTION 2:**

Hi [name],

At [Global Perspectives / Global Patients / Global Expert] we are looking for adults to participate in an upcoming paid interview study in [the US / the UK / Germany / Canada / Italy] regarding COVID-19.

Eligible participants will be invited to attend a telephone or web-assisted interview that will last approximately 60 minutes in total. Participants will be compensated with [100 USD / 65 GBP / 90 EUR / 80 CAD / 80 EUR] for their time.

As a moderator of a [social media/group/page], I am hoping that you would help me take this information to those who might be interested by sharing it or letting me post it in the group you moderate.

If you have any questions, please contact me at [Contact details].

**1.5) SOCIAL MEDIA POST LONG VERSION:**

Good [morning/afternoon/evening],

My name is [recruiter’s name] and I work for [Global Perspectives / Global Patients / Global Expert], where we look for people to take part in scientific studies.

We are looking for adults in [the US / the UK / Germany / Canada / Italy] to participate in an upcoming paid interview study.

The study will consist of a 60min telephone or web-assisted interview. These interviews are designed to explore attitudes, perceptions, and preferences in relation to COVID-19 vaccines.

We appreciate the time that people invest in our studies, and, for this reason, we will offer an incentive of [100 USD / 65 GBP / 90 EUR / 80 CAD / 80 EUR] for taking part in the interview as a token of appreciation.

If you are interested in participating or would like more information about the study, please contact [Contact details].

Many thanks for reading and wishing you a lovely day!

**1.6)** **SOCIAL MEDIA POST SHORT VERSION – OPTION 1:**

Hi,

At [Global Perspectives / Global Patients / Global Expert] we are looking for adults to participate in an upcoming paid interview study in [the US / the UK / Germany / Canada/ Italy].

Eligible participants will be invited to attend a telephone or web-assisted interview that will last approximately 60 minutes in total. Participants will be compensated with [100 USD / 65 GBP / 90 EUR / 80 CAD / 80 EUR] for their time.

If you are interested in participating or would like more information about the study, please contact [Contact details].

**1.7)** **SOCIAL MEDIA POST SHORT VERSION – OPTION 2:**

Hi,

At [Global Perspectives / Global Patients / Global Expert] we are looking for adults to participate in an upcoming paid interview study in [the US / the UK / Germany / Canada] regarding COVID-19.

Eligible participants will be invited to attend a telephone or web-assisted interview that will last approximately 60 minutes in total. Participants will be compensated with [100 USD / 65 GBP / 90 EUR / 80 CAD] for their time.

If you are interested in participating or would like more information about the study, please contact [Contact details].

**1.8) DIRECT MESSAGE ON SOCIAL MEDIA – LONG VERSION:**

Good [morning/afternoon/evening],

My name is [recruiter’s name] and I work for [Global Perspectives / Global Patients / Global Expert], where we look for people to take part in scientific studies.

We are looking for adults in [the US / the UK / Germany / Canada / Italy] to participate in an upcoming paid interview study.

The study will consist of a 60min telephone or web-assisted interview. These interviews are designed to explore attitudes, perceptions, and preferences in relation to COVID-19 vaccines.

We appreciate the time that people invest in our studies, and, for this reason, we will offer an incentive of [100 USD / 65 GBP / 90 EUR / 80 CAD/ 80 EUR] for taking part in the interview as a token of appreciation.

If you are interested in participating or would like more information about the study, please let me know by Facebook or via email at [Contact details].

Many thanks in advance!

**1.9) DIRECT MESSAGE ON SOCIAL MEDIA – SHORT VERSION – OPTION 1:**

Hi [name],

At [Global Perspectives / Global Patients / Global Expert] we are looking for adults to participate in an upcoming paid interview study in [the US / the UK / Germany / Canada / Italy].

The study consists of a telephone or web-assisted interview that will last approximately 60 minutes in total. Eligible participants will be compensated with [100 USD / 65 GBP / 90 EUR / 80 CAD / 80 EUR] for their time.

If you are interested or would like to get more information, please contact me at [Contact details].

**1.10) DIRECT MESSAGE ON SOCIAL MEDIA – SHORT VERSION – OPTION 2:**

Hi [name],

At [Global Perspectives / Global Patients / Global Expert] we are looking for adults to participate in an upcoming paid interview study in [the US / the UK / Germany / Canada / Italy] regarding COVID-19.

The study consists of a telephone or web-assisted interview that will last approximately 60 minutes in total. Eligible participants will be compensated with [100 USD / 65 GBP / 90 EUR / 80 CAD / 80 EUR] for their time.

If you are interested or would like to get more information, please contact me at [Contact details].

**1.11) SOCIAL MEDIA FLYER – OPTION 1:**

**COVID-19 Vaccines**

We currently have a study in [the US / the UK / Germany / Canada / Italy] to explore attitudes, perceptions, and preferences in relation to COVID-19 vaccines

60-minute interview

Incentive of [100 USD / 65 GBP / 90 EUR / 80 CAD / 80 EUR] as a token of appreciation

For more information please contact: [Contact details]

**1.12) SOCIAL MEDIA FLYER – OPTION 2:**

**COVID-19 Study**

We currently have a study in [the US / the UK / Germany / Canada / Italy] regarding COVID-19

60-minute interview

Incentive of [100 USD / 65 GBP / 90 EUR / 80 CAD / 80 EUR] as a token of appreciation

For more information please contact: [Contact details]

**1.13) SUPPORT GROUP**

Good [morning/afternoon/evening],

My name is [recruiter’s name] and I work for [Global Perspectives / Global Patients / Global Expert], where we look for people to take part in scientific studies.

We are looking for adults in [the US / the UK / Germany / Canada / Italy] to participate in an upcoming paid interview study.

The study will consist of a 60min telephone or web-assisted interview. These interviews are designed to explore attitudes, perceptions, and preferences in relation to COVID-19 vaccines.

We appreciate the time that participants invest in our studies and as a token of our appreciation will remunerate them [100 USD / 65 GBP / 90 EUR / 80 CAD / 80 EUR] for taking part in the interview. Collaboration is entirely voluntary, and results will remain anonymous and confidential.

If you are interested in referring people for this study or would like more information about the study, please contact **[Contact details]**.

**1.15) INVITATION TO DATABASE MEMBERS – OPTION 2:**

Hi there!

Thank you so much for being part of [Global Patients/local recruiter’s database], we hope you are safe and sound!

I am contacting you to let you know about a new study for which we are looking for adults.

Our study consists of a 60-minute telephone or web-assisted interview, for which you would be granted [100 USD / 65 GBP / 90 EUR / 80 CAD / 80 EUR]. These interviews are designed to explore attitudes, perceptions, and preferences in relation to COVID-19 vaccines.

Please let me know if you are interested and I will be happy to provide further details.

Looking forward to hearing from you.

**PHASE 2: ONLINE SURVEY**

**2.1) ONLINE PANEL - OPTION 1:**

**Topic:** Healthcare

**Full incentive:** Panel points

**Length:** 30 minutes

**Details:** Preference survey on COVID-19 vaccines

**LET’S BEGIN**

**2.2) ONLINE PANEL - OPTION 2:**

Let your voice be heard!

A new survey is waiting for you, and [panel points] to collect too.

Here's a new opportunity to share your thoughts and opinions with big brands to influence the future.

Today we'd like you to participate in this survey for the chance to collect [panel points] reward.

Click below, contribute and collect [panel points] today.

LOG IN

Please add [email address] to your address book to ensure you receive our emails.

Please note this address is for delivery purposes only.

To contact us please click here.

You received this email because you (or someone in your household) has registered with [panel points].

If you wish to be removed from the [panel points] panel, please click here

**2.3) ONLINE PANEL - OPTION 3:**

Hi [First Name],

[Company] invites you to participate in an online survey. No personally identifiable information such as email or address is required. Please contact [email address] if you have any issues with the survey.

- Survey Length: 30 minutes
- Points: [panel points] uploaded automatically into your account if you qualify and complete the survey
- *Remember every attempt is an entry in our weekly drawing for [panel points] points

If you would like to participate, click here or copy and paste the below URL into a new web browser window:

[URL link]

Thanks from the [company] team!

*This email was sent to [email address] because you signed up at [web address] to participate in research. If you have changed your mind, please unsubscribe here or reply to this email with ‘unsubscribe’ in the subject line and you will be removed within 5 business days.*

**2.4) ONLINE PANEL – OPTION 4:**

**SURVEY ID**

**SURVEY LENGTH** 30 min

**REWARD** [Panel points]

**SURVEY TOPIC** Vaccines

**Hello [name],**

We have selected a new survey for you today matching your profile. The survey is about vaccinations which would take around 30 min of your time.

We are looking to speak with general public through this survey

We would appreciate your honest and thoughtful opinion in this survey. If you are interested in sharing your opinion and get acknowledged, take the survey today and earn [AMOUNT] for your successful participation.

**Please click on the button to start the survey.**

Start Survey

If the above button doesn't work, please copy and paste the below link into your browser and hit enter.

[URL link]

**We value your opinion and look forward to hearing from you. Thank you for your time!**

Warm Regards,

Survey Team

Instructions: -

1.) If you face any technical difficulty while completing the survey or have any questions regarding this survey, you can send an e-mail to [email address] mentioning your panelist ID [panelist ID] and the survey ID [survey ID] in the subject line of the email.

2.) You will earn reward only if you answer all the questions and qualify the survey.

3.) You can check your earned amount in your account under My Rewards tab and redeem the same as per the redemption policy.

4.) We set quality check questions in the survey, kindly read each question carefully and choose the answer that best describes your opinion.

5.) Your survey URL expires as soon as you click on it, kindly try to complete the survey all at once. If you disconnect the survey after answering few questions, you won't be able to participate in the same survey again.

6.) Please activate JavaScript and enable cookies in your internet browser to access all kind of surveys.

You are receiving this email because you are a registered member of [company] Community. If you do not wish to receive any future email from [company], please click here to de-activate your account. As soon as you unsubscribe from [company], your account will be deleted within 72 hours and you will stop receiving emails from [company]. If you face any technical difficulty with the website, please reach out to our helpdesk team at [email address].

**2.5) Recruitment ANNOUNCEMENT for a Survey Study – OPTION 1:**

[Global Perspectives / Global Patients / Global Expert] is currently looking for adults to participate in an upcoming paid survey study in [the US / the UK / Germany / Canada / Italy].

The purpose of this study is to better understand attitudes, perceptions, and preferences in relation to COVID-19 vaccines. Eligible participants will be invited to complete an online survey that will last approximately 30 minutes in total. Participants will be compensated [AMOUNT] for their time.

If you are interested in participating or would like more information about the study, please contact [Contact details].

**2.6) Recruitment ANNOUNCEMENT for a Survey Study – OPTION 2:**

[Global Perspectives / Global Patients / Global Expert] is currently looking for adults to participate in an upcoming paid survey study in [the US / the UK / Germany / Canada / Italy] regarding COVID-19.

Eligible participants will be invited to complete an online survey that will last approximately 30 minutes in total. Participants will be compensated [AMOUNT] for their time.

If you are interested in participating or would like more information about the study, please contact [Contact details].

**2.7) TO ADMINS ON SOCIAL MEDIA LONG VERSION:**

Good [morning/afternoon/evening],

My name is [recruiter’s name] and I work for [Global Perspectives / Global Patients / Global Expert], where we look for people to take part in scientific studies.

We are looking for adults in [the US / the UK / Germany / Canada / Italy] to participate in an upcoming paid survey study.

The reason for my message is to know if you, as the [Group Administrator/Group Moderator] on [Social Media Channel], would allow me to post a recruitment message on the group wall in case there is someone interested in participating.

The study will consist of a 30min online survey. This survey is designed to explore attitudes, perceptions, and preferences in relation to COVID-19 vaccines.

We appreciate the time that participants invest in our studies and, for this reason, will offer an incentive of [AMOUNT]. for taking part in the survey as a token of appreciation.

If you need more information, please do not hesitate to contact me via Facebook or email at [Contact details].

Thank You!

**2.8)** **TO ADMINS ON SOCIAL MEDIA SHORT VERSION – OPTION 1:**

Hi [name],

At [Global Perspectives / Global Patients / Global Expert] we are looking for adults to participate in an upcoming paid survey study in [the US / the UK / Germany / Canada / Italy].

Eligible participants will be invited to complete an online survey that will last approximately 30 minutes in total. Participants will be compensated with [AMOUNT] for their time.

As a moderator of a [social media/group/page], I am hoping that you would help me take this information to those who might be interested by sharing it or letting me post it in the group you moderate.

If you have any questions, please contact me at [Contact details].

**2.9)** **TO ADMINS ON SOCIAL MEDIA SHORT VERSION – OPTION 2:**

Hi [name],

At [Global Perspectives / Global Patients / Global Expert] we are looking for adults to participate in an upcoming paid survey study in [the US / the UK / Germany / Canada / Italy] regarding COVID-19.

Eligible participants will be invited to complete an online survey that will last approximately 30 minutes in total. Participants will be compensated with [AMOUNT] for their time.

As a moderator of a [social media/group/page], I am hoping that you would help me take this information to those who might be interested by sharing it or letting me post it in the group you moderate.

If you have any questions, please contact me at [Contact details].

**2.10) SOCIAL MEDIA POST LONG VERSION:**

Good [morning/afternoon/evening],

My name is [recruiter’s name] and I work for [Global Perspectives / Global Patients / Global Expert], where we look for people to take part in scientific studies.

We are looking for adults in [the US / the UK / Germany / Canada / Italy] to participate in an upcoming paid survey study.

The study will consist of a 30min online survey. This survey is designed to explore attitudes, perceptions, and preferences in relation to COVID-19 vaccines.

We appreciate the time that people invest in our studies, and, for this reason, we will offer an incentive of [AMOUNT] for taking part in the survey as a token of appreciation.

If you are interested in participating or would like more information about the study, please contact [Contact details].

Many thanks for reading and wishing you a lovely day!

**2.11)** **SOCIAL MEDIA POST SHORT VERSION – OPTION 1:**

Hi,

At [Global Perspectives / Global Patients / Global Expert] we are looking for adults to participate in an upcoming paid survey study in [the US / the UK / Germany / Canada / Italy].

Eligible participants will be invited to complete an online survey that will last approximately 30 minutes in total. Participants will be compensated with [AMOUNT] for their time.

If you are interested in participating or would like more information about the study, please contact [Contact details].

**2.12)** **SOCIAL MEDIA POST SHORT VERSION – OPTION 2:**

Hi,

At [Global Perspectives / Global Patients / Global Expert] we are looking for adults to participate in an upcoming paid survey study in [the US / the UK / Germany / Canada / Italy] regarding COVID-19.

Eligible participants will be invited to complete an online survey that will last approximately 30 minutes in total. Participants will be compensated with [AMOUNT] for their time.

If you are interested in participating or would like more information about the study, please contact [Contact details].

**2.13) DIRECT MESSAGE ON SOCIAL MEDIA – LONG VERSION:**

Good [morning/afternoon/evening],

My name is [recruiter’s name] and I work for [Global Perspectives / Global Patients / Global Expert], where we look for people to take part in scientific studies.

We are looking for adults in [the US / the UK / Germany / Canada / Italy] to participate in an upcoming paid survey study.

The study will consist of a 30min online survey. This survey is designed to explore attitudes, perceptions, and preferences in relation to COVID-19 vaccines.

We appreciate the time that people invest in our studies, and, for this reason, we will offer an incentive of [AMOUNT] for taking part in the survey as a token of appreciation.

If you are interested in participating or would like more information about the study, please let me know by Facebook or via email at [contact details].

Many thanks in advance!

**2.14) DIRECT MESSAGE ON SOCIAL MEDIA – SHORT VERSION – OPTION 1:**

Hi [name],

At [Global Perspectives / Global Patients / Global Expert] we are looking for adults to participate in an upcoming paid survey study in [the US / the UK / Germany / Canada].

The study consists of an online survey that will last approximately 30 minutes in total. Participants will be compensated with [AMOUNT] for their time.

If you are interested or would like to get more information, please contact me at [Contact details].

**2.15) DIRECT MESSAGE ON SOCIAL MEDIA – SHORT VERSION – OPTION 2:**

Hi [name],

At [Global Perspectives / Global Patients / Global Expert] we are looking for adults to participate in an upcoming paid survey study in [the US / the UK / Germany / Canada / Italy] regarding COVID-19.

The study consists of an online survey that will last approximately 30 minutes in total. Participants will be compensated with [AMOUNT] for their time.

If you are interested or would like to get more information, please contact me at [Contact details].

**1.16) SOCIAL MEDIA FLYER – OPTION 1:**

**COVID-19 Vaccines**

We currently have a study in [the US / the UK / Germany / Canada / Italy] to explore attitudes, perceptions, and preferences in relation to COVID-19 vaccines

30-minute online survey

Incentive of [AMOUNT] as a token of appreciation

For more information please contact: [Contact details]

**1.17) SOCIAL MEDIA FLYER – OPTION 2:**

**COVID-19 Study**

We currently have a study in [the US / the UK / Germany / Canada / Italy] regarding COVID-19

30-minute online survey.

Incentive of [AMOUNT] as a token of appreciation

For more information please contact: [Contact details]

**2.18) SUPPORT GROUP:**

Good [morning/afternoon/evening],

My name is [recruiter’s name] and I work for [Global Perspectives / Global Patients / Global Expert], where we look for people to take part in scientific studies.

We are looking for adults in [the US / the UK / Germany / Canada / Italy] to participate in an upcoming paid survey study.

The study will consist of a 30-minute online survey. This survey is designed to explore attitudes, perceptions, and preferences in relation to COVID-19 vaccines.

We appreciate the time that participants invest in our studies and as a token of our appreciation will remunerate them [AMOUNT] for taking part in the survey. Collaboration is entirely voluntary, and results will remain anonymous and confidential.

If you are interested in referring people for this study or would like more information about the study, please contact [Contact details].

**2.20) INVITATION TO DATABASE MEMBERS – OPTION 2:**

Hi there!

Thank you so much for being part of [Global Patients/local recruiter’s database], we hope you are safe and sound!

I am contacting you to let you know about a new study for which we are looking for adults.

Our study consists of a 30-minute online survey, for which you would be granted [AMOUNT]. This survey is designed to explore attitudes, perceptions, and preferences in relation to COVID-19 vaccines.

Please let me know if you are interested and I will be happy to provide further details.

Looking forward to hearing from you.
